# Molecular Etiology Disclosed by Array CGH in Patients With Silver–Russell Syndrome or Similar Phenotypes

**DOI:** 10.3389/fgene.2019.00955

**Published:** 2019-10-15

**Authors:** Milena Crippa, Maria Teresa Bonati, Luciano Calzari, Chiara Picinelli, Cristina Gervasini, Alessandra Sironi, Ilaria Bestetti, Sara Guzzetti, Simonetta Bellone, Angelo Selicorni, Alessandro Mussa, Andrea Riccio, Giovanni Battista Ferrero, Silvia Russo, Lidia Larizza, Palma Finelli

**Affiliations:** ^1^Research Laboratory of Medical Cytogenetics and Molecular Genetics, IRCCS Istituto Auxologico Italiano, Milan, Italy; ^2^Department of Medical Biotechnology and Translational Medicine, University of Milan, Milan, Italy; ^3^Clinic of Medical Genetics, IRCCS Istituto Auxologico Italiano, Milan, Italy; ^4^Medical Genetics, Department of Health Sciences, University of Milan, Milan, Italy; ^5^Division of Pediatrics, Department of Health Sciences, University of Piemonte Orientale, Novara, Italy; ^6^Department of Pediatrics, ASST-Lariana, Como, Italy; ^7^Department of Pediatric and Public Health Sciences, University of Turin, Turin, Italy; ^8^Department of Environmental, Biological and Pharmaceutical Sciences and Technologies, University of Campania “Luigi Vanvitelli,” Caserta, Italy; ^9^Institute of Genetics and Biophysics “Adriano Buzzati-Traverso,” Consiglio Nazionale delle Ricerche (CNR), Naples, Italy

**Keywords:** Silver–Russell syndrome, Netchine–Harbison clinical scoring system, array CGH, pathogenic CNVs, differential diagnosis

## Abstract

**Introduction:** Silver–Russell syndrome (SRS) is an imprinting disorder primarily caused by genetic and epigenetic aberrations on chromosomes 11 and 7. SRS is a rare growth retardation disorder often misdiagnosed due to its heterogeneous and non-specific clinical features. The Netchine–Harbison clinical scoring system (NH-CSS) is the recommended tool for differentiating patients into clinical SRS or unlikely SRS. However, the clinical diagnosis is molecularly confirmed only in about 60% of patients, leaving the remaining substantial proportion of SRS patients with unknown genetic etiology.

**Materials and Methods:** A cohort of 34 Italian patients with SRS or SRS-like features scored according to the NH-CSS and without any SRS-associated (epi)genetic alterations was analyzed by high-resolution array-based comparative genomic hybridization (CGH) in order to identify potentially pathogenic copy number variants (CNVs).

**Results and Discussion:** In seven patients, making up 21% of the initial cohort, five pathogenic and two potentially pathogenic CNVs were found involving distinct genomic regions either previously associated with growth delay conditions (1q24.3-q25.3, 17p13.3, 17q22, and 22q11.2-q11.22) and with SRS spectrum (7p12.1 and 7p15.3-p14.3) or outlined for the first time (19q13.42), providing a better definition of reported and as yet unreported SRS overlapping syndromes. All the variants involve genes with a defined role in growth pathways, and for two genes mapping at 7p, *IGF2BP3* and *GRB10*, the association with SRS turns out to be reinforced. The deleterious effect of the two potentially pathogenic variants, comprising *GRB10* and *ZNF331* genes, was explored by targeted approaches, though further studies are needed to validate their pathogenic role in the SRS etiology. In conclusion, we reconfirm the utility of performing a genome-wide scan to achieve a differential diagnosis in patients with SRS or similar features and to highlight novel chromosome alterations associated with SRS and growth retardation disorders.

## Introduction

Silver–Russell syndrome (SRS; Online Mendelian Inheritance in Man (OMIM) #180860, also known as Russell–Silver syndrome, RSS) is an imprinting disorder with a broad clinical and molecular heterogeneity. The clinical diagnosis of SRS is difficult, as many of its features are non-specific and overlap with those of several other congenital syndromes; moreover, the severity of clinical presentation can be highly variable (Wakeling et al., 2017). As recommended by the recently published first international consensus statement, the Netchine–Harbison clinical scoring system (NH-CSS) (Azzi et al., 2015) should be applied for SRS diagnosis (Wakeling et al., 2017). The NH-CSS includes six key features: being born small for gestational age (SGA) (birth weight/length ≤ −2 standard deviation (SD)), postnatal growth retardation (weight/length ≤ −2 SD), relative macrocephaly at birth (head circumference ≥ 1.5 SD above birth weight and/or length SD), body asymmetry (leg length discrepancy ≥ 0.5 cm or <0.5 cm with at least two other asymmetrical body parts), feeding difficulties and/or low body mass index (BMI) (BMI ≤ −2 SD at 24 months or current use of a feeding tube or cyproheptadine for appetite stimulation), and a protruding forehead (Azzi et al., 2015). A patient receives a diagnosis of SRS when he/she meets four or more of these six criteria (positive NH-CSS ≥4/6), while patients with a negative NH-CSS (<4/6) are generally classified as unlikely SRS (Azzi et al., 2015). However, despite the high sensitivity of this clinical score, patients scoring 4 or more criteria may have negative molecular testing and vice versa (Tümer et al., 2018). In accordance with the statement, patients meeting NH-CSS criteria including both protruding forehead and relative macrocephaly, but normal at all molecular tests, receive a diagnosis of clinical SRS. The two primary molecular causes of SRS are hypomethylation of the distal imprinting control region *H19/IGF2*:IG-DMR (ICR1) at 11p15.5, identified in about 30–60% of patients, and maternal uniparental disomy of chromosome 7 (upd(7)mat), detected in about 5–10% of patients (Wakeling et al., 2017). In addition chromosome 14q32.2 imprinting defects, underpinning Temple syndrome (TS; OMIM#616222), represent an alternative molecular diagnosis to SRS, as numerous molecularly confirmed TS patients (70–72.7%) were found to fulfill the NH-CSS criteria (Kagami et al., 2017; Geoffron et al., 2018). Further rare genetic alterations, that is, point mutations in imprinted genes at 11p15 or in other disease-causing genes, and non-recurrent copy number variants (CNVs) affecting different chromosome regions have been detected in a small number of SRS patients (Bruce et al., 2010; Lin et al., 2010; Spengler et al., 2012; Fuke et al., 2013; Inoue et al., 2017; Meyer et al., 2017; Sachwitz et al., 2017; Wakeling et al., 2017; Tumer et al., 2018).

However, in a significant proportion of SRS patients (up to 30%), the molecular etiology remains unknown, and SRS represents primarily a clinical diagnosis. Genome-wide approaches such as chromosome microarray analysis and next-generation sequencing in patients referred for SRS testing have been shown to enhance the molecular diagnosis, often unveiling SRS overlapping disorders (Bruce et al., 2010; Spengler et al., 2012; Fuke et al., 2013; Inoue et al., 2017; Meyer et al., 2017; Sachwitz et al., 2017; Tumer et al., 2018). To this purpose, and to identify novel rearrangements and genes related to SRS, we performed high-resolution array-based comparative genomic hybridization (aCGH) analysis on a group of 34 patients, fulfilling and not fulfilling the NH-CSS criteria, without a molecular diagnosis.

## Materials and Methods

### Study Cohort

The study population consisted of 34 Italian patients (19 males and 15 females) born SGA and/or with postnatal growth retardation referred as SRS for diagnostic testing and found negative for *H19/IGF2*:IG-DMR LOM and upd(7)mat.

Clinical information was collected by clinicians at different hospitals who participated in the study, using the same clinical datasheet. SRS clinical scoring was based on the NH-CSS (Azzi et al., 2015) with exceptions when postnatal growth and BMI were assessed at different ages, as performed by Meyer et al. (2017). NH-CSS was assessed in all but two patients due to incomplete clinical data. Neonatal SD was calculated according to the Italian Neonatal Study (INeS) charts (http://www.inescharts.com/), whereas postnatal SD was assessed according to Cacciari et al. (2006). Of the 32 patients, 21(66%) satisfied NH-CSS, 19 out of 21 with a score ≥4/6 and two with a score ≥4/5 as the macrocephaly at birth was missing in the latter. Among the patients fulfilling the NH-CSS criteria, 42% (8/19) showed both protruding forehead and relative macrocephaly at birth, consistent with clinical SRS. The remaining 11 patients turned out to have a negative clinical score but were included in the study since they presented other clinical features suggestive of SRS, for example, triangular face and/or micrognathia and/or fifth finger clinodactyly; in detail, seven patients matched three NH-CSS criteria, and four presented only two clinical items. Furthermore, in all patients with a positive clinical score for SRS, aberrant methylation/uniparental disomy of the imprinted locus *MEG3*-DMR in 14q32 was ruled out. **Table 1** reports the clinical data and the presence/absence of the various NH-CSS criteria in our whole SRS cohort and its two clinical subgroups. **Table 1** also provides the frequency of each clinical finding of the scoring system in our clinically confirmed SRS subgroup compared with that in literature, showing a substantial level of consistency between the two cohorts.

**Table 1:** Distribution of our SRS cohort, negative to 11p15 defects and upd(7)mat, according to NH-CSS criteria and comparison with literature.

	SRS study cohort 11p15 LOM and upd(7)mat excluded	SRS study cohort NH-CSS <4 group	SRS study cohort NH-CSS ≥ 4 group	Reference SRS cohort NH-CSS ≥4,11p15 LOM and upd(7)mat excluded (Wakeling et al., 2017)	Reference SRS cohort 11p15 LOM and upd(7)mat and/or NH-CSS ≥4 (Wakeling et al., 2017)
**Patient no. and (%)**	34 #	11 (34%)	21 (66%)	–	–
**Age at examination (years)**	4.0 ± 3.7	5.8 ± 4.2	3.0 ± 3.0	–	–
***NH-CSS factors% and (patients no)***					
**Birth weight/height ≤ −2 SD**	64% (21/33)	54% (6/11)	71% (15/21)	85.7% (12/14)	91.7% (55/60)
**Relative macrocephaly at birth §**	36% (10/28)	22% (2/9)	42% (8/19)	55.4% (31/56)	85.7% (179/209)
**Postnatal growth failure ‡**	91% (30/33)	82% (9/11)	95% (20/21)	86.8%* (72/83)	84.2% * (267/317)
**Protruding forehead**	80% (25/31)	50% (5/10)	95% (20/21)	77.6% (59/76)	88.1% (177/201)
**Body asymmetry**	41% (13/32)	9% (1/11)	57% (12/21)	39.5% (62/157)	57.3% (/271473)
**Feeding difficulties and/or BMI ≤ −2 SD**	79% (26/33)	54% (6/11)	95% (20/21)	54.8%** (40/73)	70.4% ** (216/307)
***Additional signs% and (patients no)***					
**Preterm birth**	42% (13/31)	55% (5/9)	38% (8/21)	n.k.	
**Triangular face**	84% (26/31)	90% (9/10)	80% (16/20)	98.7% (73/74)	93.9% (154/164)
**Micrognathia**	55% (17/31)	60% (6/10)	50% (10/20)	56% (5/9)	61.7% (71/115)
**Downturned mouth**	53% (16/30)	30% (3/10)	65% (13/20)	39.1% (9/23)	47.7% (84/176)
**Low-set and/or posteriorly rotated ears**	30% (9/30)	40% (4/10)	25% (5/20)	35.9% (28/78)	49.3% (131/266)
**Clinodactyly of 5th finger**	55% (17/31)	50% (5/10)	57% (12/21)	77.8% (63/81)	74.6% (238/319)
**2nd or 3rd toe syndactyly**	16% (5/31)	30% (3/10)	9.5% (2/21)	16% (12/75)	29.9% (79/264)
**Low muscle mass**	28% (9/32)	18% (2/11)	33% (7/21)	34.8% (8/23)	56.3% (58/103)
**Male genital abnormalities**	44% (8/18)	50% (3/6)	41.6% (5/12)	37.5% (3/8)	40% (34/85)
**Psychomotor delay**	33% (9/27)	45% (5/11)	25% (4/16)	41.3%*** (26/63)	36.6%*** (93/254)
**Speech delay**	28.5% (6/21)	33% (3/9)	17% (3/12)	38.5% (20/52)	39.7% (75/189)

^#^Incomplete clinical evaluation in 2 patients; ^§^Head circumference SD ≥1.5 higher than birth weight or length SD;

^‡^Weight and/or length ≤ −2 SD at the age of evaluation; *Only postnatal height considered **Only feeding difficulties considered, ***Only motor delay considered; n.k., not known; SRS, Silver–Russell syndrome; NH-CSS, Netchine– Harbison clinical scoring system.

The study was approved by the ethical committee of IRCSS Istituto Auxologico Italiano, and written informed consent was obtained from the parents for publication of the study and any accompanying images.

### aCGH and CNV Analysis

aCGH analysis was performed using the high-resolution Human Genome CGH Microarray Kits, namely, microarray formats 244K (median resolution of 27 kb) (18 patients) and 2 × 400K (median resolution of 16 kb) (16 patients) (Agilent Technologies, Palo Alto, CA) in accordance with the manufacturer’s instructions. Data were then extracted and analyzed for copy number changes using Agilent CytoGenomics 3.0.

Detected CNVs, identified by at least three consecutive aberrant probes, were compared with the Database of Genomic Variants (DGV) (http://projects.tcag.ca/variation/, release March 2016), to exclude common copy number polymorphisms (frequency >1%). A CNV was classified as rare if unreported or reported at a very low frequency («1%) according to the DGV. Inherited rare CNVs were considered automatically validated, whereas the *de novo* CNVs (or with an unknown inheritance), detected by a number of oligonucleotide consecutive probes ≤5, were validated using quantitative polymerase chain reaction (qPCR).

The establishment of CNV pathogenicity was made considering inheritance, familial segregation, and the consultation of public databases, to assess (i) whether the CNVs overlap with common microdeletion/microduplication syndromes, (ii) the CNVs’ gene content, and (iii) the clinical information of patients with similar CNVs. Specifically, the following public databases were consulted: University of California Santa Cruz (UCSC) (http://genome.ucsc.edu/, release February 2009), OMIM (www.ncbi.nlm.nih.gov/OMIM), International Standards for Cytogenomic Arrays (ISCA) (https://www.clinicalgenome.org/, update November 2012), DECIPHER v9.10 (http://www.sanger.ac.uk/PostGenomics/decipher/, last access December 2016) (Bragin et al., 2014), and PubMed (https://www.ncbi.nlm.nih.gov/pubmed/, last access March 2016). The involvement of the identified genes in cell cycle, developmental process, and/or cell growth pathways was investigated using Entrez Gene (https://www.ncbi.nlm.nih.gov/gene) and potential interaction with already-associated SRS genes was assessed with STRING, a tool for Protein–Protein Interaction Networks (https://string-db.org/). Finally, the evaluation of imprinting status of the genes involved in the regions was carried out using the Geneimprint database (http://www.geneimprint.com/site/genes-by-species), and a search for murine knockout phenotypes with growth impairment was made with the Mouse Genome Informatics (MGI) database (Eppig et al., 2012).

The guidelines suggested by Miller et al. (2010) and successively by the American College of Medical Genetics (Kearney et al., 2011) were followed for CNV classification with minor modifications.

### Microsatellite Analysis

To assess the parental origin of the structural variant at 7p15.3p14.3 identified in patient 20, microsatellite analysis was performed using 8 STR markers, that is, D7S513, D7S493, D7S673, D7S2525, D7S2449, D7S516, D7S2496, and D7S526, within chromosome 7p. All fluorescent PCR amplicons were run on capillary electrophoresis using the 3500 Genetic Analyzer (Applied Biosystems). Data analysis and genotyping were carried out by the Genemapper software (Applied Biosystems), matching parents to proband transmission.

### Real-Time Reverse Transcriptase (RT)-PCR

Quantitative gene expression analysis was performed in patients 18 and 20 to explore a potential dysregulation in gene expression mediated by the CNVs identified in the patients. Total RNA of patients, their parents, and 10 healthy controls were collected using Tempus Blood RNA tubes (Thermo Fisher Scientific, Waltham, MA), isolated using the Tempus Spin RNA Isolation kit (Thermo Fisher Scientific), and reverse transcribed using the High-Capacity cDNA Reverse Transcription kit (Thermo Fisher Scientific). Quantitative real-time RT-PCR, based on the TaqMan methodology, was performed using an ABI PRISM 7700 Sequence Detection System (Applied Biosystems, Foster City, CA). In patient 18, the amounts of *GRB10* mRNA were calculated using the 2^−ΔΔCt^ method (Livak and Schmittgen, 2001), with *GAPDH*, *HMBS*, *RPRPL0*, and *TBP* as the endogenous-normalizing genes, while in patient 20 the amounts of *IGF2BP3* mRNA were calculated with the same method, using the *GAPDH*, *GUSB*, and *TBP* as the endogenous-normalizing genes. All assays were provided by Thermo Fisher Scientific (TaqMan Gene Expression Assays: ID# Hs00959293_m1, GRB10; Hs00946580_m1, IGF2BP3; Hs99999905_m1, GAPDH; Hs00939627_m1, GUSB; Hs00609297_m1, HMBS; Hs99999902_m1, RPLP0; and Hs99999910_m1, TBP). Real-time data were analyzed using the RQ Manager 1.2 software (Applied Biosystems). For each gene of interest, we established the proper range of gene expression in 10 healthy controls by calculating the mean value ± 2 SD. If the expression level of the CNV carrier was out of the control range, a likely dysregulation of the index gene mediated by the CNV was inferred.

### Quantitative PCR

qPCR analysis was performed on genomic DNA of patients 8 and 20 using SYBR Green methodology (Thermo Fisher Scientific). qPCR was used in patient 8 to finely map the proximal CNV boundary at 19q13.42, and in patient 20 to validate the presence of a small *de novo* deletion at 7p21.1 detected by three aCGH probes. All amplicons were chosen within non-repeated portions of the chromosomes using Primer3 software (http://bioinfo.ut.ee/primer3-0.4.0/). A control amplicon was selected with the same parameters in the *PCNT* gene at 11q14.1. Size (approximately 60–100 bp) and *T*_m_ (60°C) were the same for all amplicons. Amplification and detection were performed on the ABI PRISM 7900HT Sequence Detection System (Applied Biosystems); thermal cycling conditions were 50°C for 2 min and 95°C for 10 min, followed by 40 cycles at 95°C for 15 s and 60°C for 1 min. Each experiment was performed in triplicate on patients, parents, and two controls. The results were acquired with the SDS v2.3 software (Applied Biosystems) and processed using RQ Manager 1.2 (Applied Biosystems). Relative quantification of the amount of DNA was obtained using the 2^−ΔΔCt^ method (Livak and Schmittgen, 2001). The primer pairs used are summarized in **Supplementary Table 1**.

### Sequencing Analysis

In patient 28, carrier of a genomic deletion involving the *TRIM37* gene, causative of the recessive disease MULIBREY nanism (OMIM#253250), the entire coding sequence, intron–exon junctions, and untranslated exons of the *TRIM37* gene (RefSeq Accession: NM_001005207) were amplified for mutation screening by PCR using the AmpliTaq Gold^®^ kit (Thermo Fisher Scientific). Sequencing was performed using the Big Dye^®^ Terminator v.3.1 Cycle Sequencing kit (Thermo Fisher Scientific). Sequences were then aligned to the human reference genome sequence (human genome assembly GRCh37/hg19) and analyzed with the ChromasPro 1.5 software (Technelysium Pty Ltd., Tewantin QLD, Australia). The primer pairs and amplification conditions are summarized in **Supplementary Table 2**.

### Pyrosequencing

Pyrosequencing analysis was performed in patients 8 and 18 to assess a perturbation in the methylation level, respectively, of *GRB10* and *ZNF331* differentially methylated regions (DMRs), possibly mediated by the structural variants identified in the patients. Sodium bisulphite conversion of DNA (500–700 ng) was performed by the EZ DNA Methylation Kit (Zymo Research Corporation, Orange, CA). PCR analysis was performed on bisulphite-treated DNA using forward and reverse primers, one of which was biotinylated.

Two assays specific for GRB10:alt-TSS-DMR, that is, fragment I (chr7:50849713-50850034, hg19) and II (chr7:50850569-50850872, hg19), and one assay specific for ZNF331:alt-TSS-DMR2 (chr19:54058016-54058229, hg19) were designed for patients 8 and 18, respectively. Pyrosequencing experiments were performed using specific sequencing primers to quantify four CpG sites for *ZNF331*, eight for *GRB10* fragment I, and six sites for *GRB10* fragment II. Quantitative DNA methylation analysis was performed using a Pyro Mark ID instrument (QIAGEN, Silicon Valley, CA) in the PSQ HS 96 System with the PyroGold SQA reagent kit (Diatech Pharmacogenetics srl, Jesi, Italy) according to the manufacturer’s instructions. Raw data were analyzed using Q-CpG software v1.0.9 (Qiagen srl), which calculates the ratio of converted C’s (T’s) to unconverted C’s at each CpG, giving the percentage of methylation.

For each sample, the methylation value represents the mean between at least two independent PCR and pyrosequencing experiments. For each assay, we established the proper range of methylation in healthy controls by calculating the mean value ± 2 SD; 11 controls for *ZNF331*, 24 controls for *GRB10* fragment I, and eight controls for *GRB10* fragment II were used.

The primer pairs and amplification conditions are summarized in **Supplementary Table 3**.

## Results

### Genomic Imbalances by aCGH and Clinical Records of the Carrier Patients

DNA samples from 34 patients with a clinical suspicion of SRS were screened by aCGH analysis. In total, in 22 of the 34 patients (65%), 40 CNVs classified as rare in the Database of Genomic Variants were detected. Of the 40 variants, 18 were deletions (ranging from 11 kb to 11 Mb), and 22 were duplications (ranging from 22.8 kb to 3 Mb). The aCGH of parental DNA revealed that in four patients, the variants were *de novo*, whereas in 15 patients, they had been inherited from an unaffected parent. Parental DNA samples were not available in three patients (**Supplementary Table 4**).

The role of CNVs in SRS-like clinical presentation was assessed by integrating the analysis of gene content, with a focus on genes involved in growth and development, with literature and database information. We categorized as pathogenic five CNVs, including four *de novo* CNVs and one inherited unmasking a recessive allele, and as potentially pathogenic (PP) two inherited CNVs (**Table 2**). All the remaining CNVs (*n* = 33) were considered either VOUS, that is, variants of unknown significance, or likely benign (LB) CNVs (**Supplementary Table 4**). All pathogenic and PP-CNVs identified in patients were submitted to the ClinVar database (http://www.ncbi.nlm.nih.gov/clinvar/, 19 December 2018, date last accessed; accession numbers SCV000863548 to SCV000863554).

**Table 2:** Genomic imbalances detected using array CGH and categorized as pathogenic (P) or potentially pathogenic (PP) variants.

Patient#	Gain/loss	CNV description according to the ISCN nomenclature^a^	Size	Inheritance	No. of affected genes	DECIPHER^b^	Genes with functions in cell cycle, developmental process, and cell growth pathways
P-CNV							
#7	Loss	arr[GRCh37] 1q24.3q25.3(172652343_183538289)x1 dn	11 Mb	*De novo*	98	13	*CENPL*, *GAS5*, *TNN*, *RFWD2*, *PAPPA2*, *BRINP2*, *RASAL2*, *ABL2*, *QSOX1*, *LHX4*, *CACNA1E*, *RGS16*, *LAMC1*
#17	Gain	arr[GRCh37] 17p13.3(1130776_1361490)x3 dn	231 kb	*De novo*	5		*TUSC5*, *YWHAE*, *CRK*
#20	Loss	arr[GRCh37] 7p15.3p14.3(23236782_30690453)x1 dn	7.5 Mb	*De novo*	80	5	*GPNMB*, *IGF2BP3*, *NPY*, *SKAP2*, *HOXA1*, *HOXA2*, *HOXA3*, *HOXA4*, *HOXA5*, *HOXA6*, *HOXA7*, *HOXA9*, *HOXA10*, *HOXA11*, *HOXA13*
#23	Loss	arr[GRCh37] 22q11.2q11.22(21808950_22963000)x1 dn	1.15 Mb	*De novo*	21	10	*UBE2L3*, *MAPK1*
#28	Loss	arr[GRCh37] 17q22(57075470_57235248)x1 mat	159.8 kb	MAT	3	1	*TRIM37*
PP-CNV							
#8	Gain	arr[GRCh37] 19q13.42(54039784_54484439)x 3 mat	445 kb	MAT	58		*ZNF331*
#18	Gain	arr[GRCh37] 7p12.1(50981149_51956510)x 3 mat	975 kb	MAT	1		(*GRB10*)

a International System for Human Cytogenomic Nomenclature (ISCN 2016).

b Number of reported patients with overlapping CNVs and prenatal and/or postnatal growth retardation.

() genes possibly deregulated by a position effect mechanism.

CGH, comparative genomic hybridization; CNV, copy number variant.

**Table 3** outlines the NH-CSS on the basis of the main clinical features at birth and at last clinical evaluation of the seven patients found to carry pathogenic (*n* = 5) and PP (*n* = 2) CNVs.

**Table 3:** Clinical data of patients from the study cohort carrying pathogenic (P) or potentially pathogenic (PP) variants.

Patient	P-CNV					PP-CNV	
	7	17	20	23	28	8	18
**NH-CS**	3/6	4/6	4/6	2/6	5/6	n.d.	4/5
**Sex**	Male	Male	Female	Female	Male	Female	Female
**Gestational age**	34	38	38+2	35	36	39	32
**Birth length(SD)**	38 cm (−2.36)	46 cm (−1.76)	43.4 cm (−2.45)	41 cm (−1.99)	n.k. (−2)	48 cm (−0.61)	38 cm (−1.51)
**Birth weight (SD)**	1087 g (−2.51)	2500 g (−1.69)	1925 g (−2.6)	1570 g (−2.01)	2060 g (−1.42)	2940 g (−0.54)	1230 g (−1.36)
**OFC at birth (SD)**	27 cm (−2.49)	33.5 cm (−0.57)	31.1 cm (−1.89)	29 cm (−2.02)	n.k. (−2)	n.k.	n.k.
**Relative macrocephalyat birth**	No	Yes	No	No	No	n.k.	n.k.
**Age at examination**	28 months	11 months	6 years 1 month	3 years	3 years 2 months	12 years 7 months	11 years
**Length (SD)**	76 cm (−3.77)	75 cm (0.19)	99.8 cm (−3.16)	85.3 cm (−2.45)	79.6 cm (−3.76)	141.5 cm (−1.95)	130 cm (−2.23)
**Weight (SD)**	8.7 kg (−4.55)	7.1 kg (−2.56)	12.1 kg (−4.43)	9.8 kg (−3.52)	8.2 kg (−6.3)	36.5 kg (−1.39)	22.5 kg (−2.9)
**BMI (SD)**	15.3 (−0.88)	12.6 (−3.78)	12.1 (−3.25)	13.47 (−1.84)	12.94 (−3.39)	18.2 (−0.66)	13.3 (−2.7)
**Relative macrocephaly**	No	Yes	Yes	No	No	n.k.	No
**Feeding difficulties**	Yes	Yes	Yes	No	Yes	n.k.	Yes
**Protruding forehead**	No	Yes	Yes	No	Yes	No	Yes
**Body asymmetry**	No	No	No	No	Yes	n.k.	Yes
**Craniofacial dysmorphisms**	Slight dolichocephaly, triangular face, downturned corners of the mouth, diastema of maxillary central incisors, fusion of upper right central and lateral incisor, slight micro-retrognathia	Triangular face, downturned mouth, thin lips	Triangular face	Triangular face, downturned mouth, thin lips, abnormal ears	(Mozzillo et al., 2016)	Triangular face, thin lips, micrognathia	Deep-set eyes, prognathism, bushy eyebrows
**Psychomotor developmental delay**	Yes, only developmental	No	Yes	No	(Mozzillo et al., 2016)	n.k.	Yes
**Other findings**	5th finger clino/brachydactyly,flat feet, delayed bone maturation, balanic hypospadias with chordae of penis and schisis of prepuce,attention deficit, poor social interaction	5th finger clino/brachydactyly, right undescended testicle, muscle hypotonia	Myopia and astigmatism, small hands with 5th finger clino/brachydactyly, small feet with short and broad halluces and symmetric skeletal anomalies, duplex collecting system of right kidney	5th finger clino/brachydactyly, 2nd and 3rd toe syndactyly	(Mozzillo et al., 2016)	n.k.	Gastroesophageal reflux, gastrointestinal abnormalities

n.k., not known; CNV, copy number variant; OFC, occipitofrontal head circumference; BMI, body mass index.

### Pathogenic Variants (P-CNVs)

**Figure 1:**
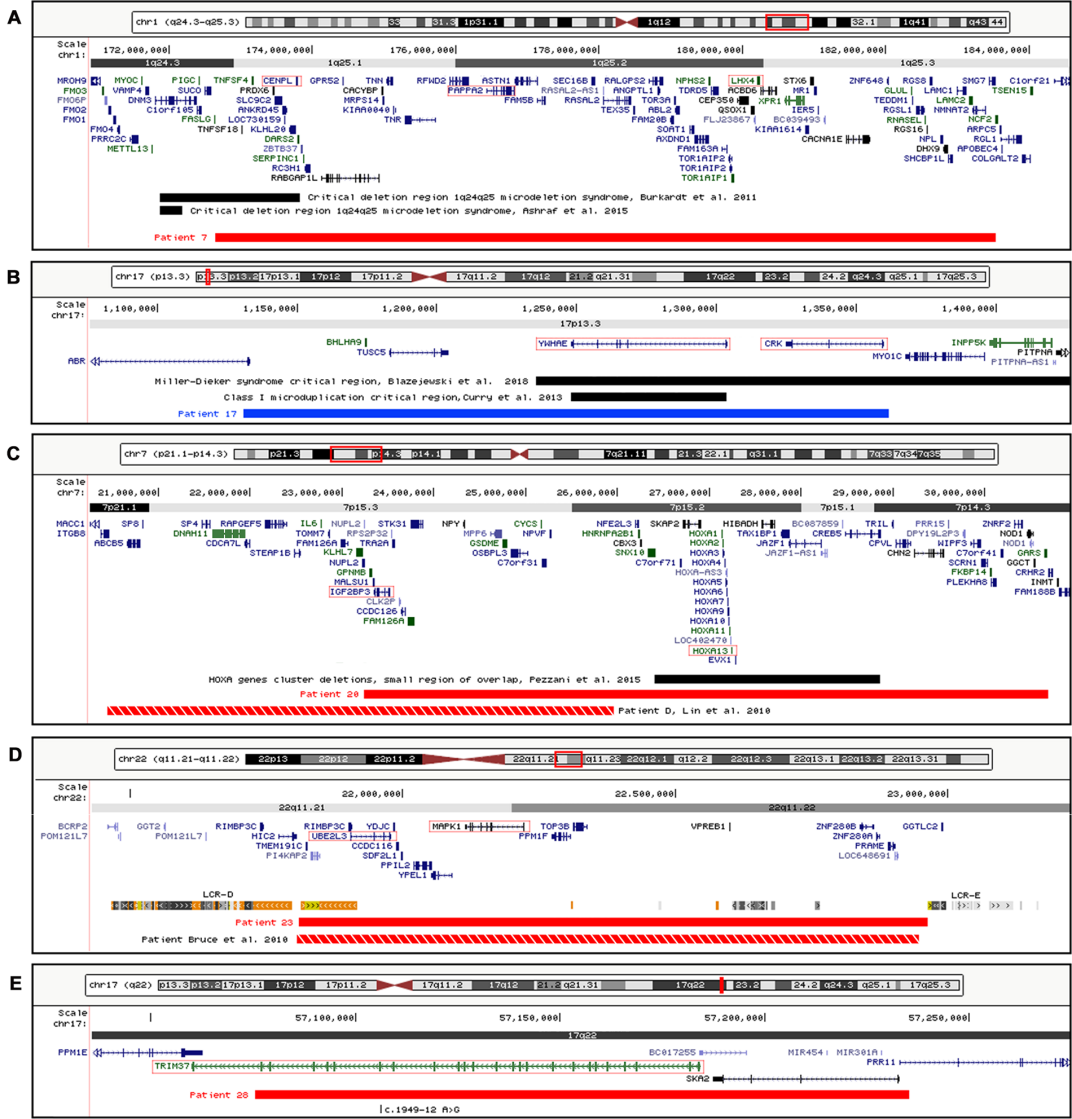
Physical map of the genomic regions containing the pathogenic CNVs identified by array CGH. **(A)** 1q24.3-q25-5 region showing the deletion detected in patient 7. **(B)** 17p13.3 region showing the duplication detected in patient 17. **(C)** 7p21.1-p14.3 region showing the deletion detected in patient 20. **(D)** 22q11.21-q11.22 region showing the deletion detected in patient 23 and the low copy repeats (LCR-E and LCR-D) surrounding the deletion bkps. **(E)** 17q22 region showing the deletion and the mutation c.1949-12 A > G involving *TRIM37* gene detected in patient 28. The RefSeq genes are depicted in dark blue, and the OMIM disease genes in green; the disease critical regions are indicated by black bars, the deletions by red bars, the duplications by blue bars, the deletions of previously reported SRS patients by red-white bars, and the candidate genes by red boxes. The images are a modification of a version obtained from the UCSC Genome Browser (human genome assembly GRCh37/hg19). CNVs, copy number variants; CGH, comparative genomic hybridization; OMIM, Online Mendelian Inheritance in Man; SRS, Silver–Russell syndrome; UCSC, University of California Santa Cruz.

Overall, chromosomal imbalances with obvious pathogenicity could be identified in five patients (7, 17, 20, 23, and 28) (**Table 2**). **Figure 1** shows the genomic regions containing the pathogenic CNVs of the carrier patients.

**Figure 2:**
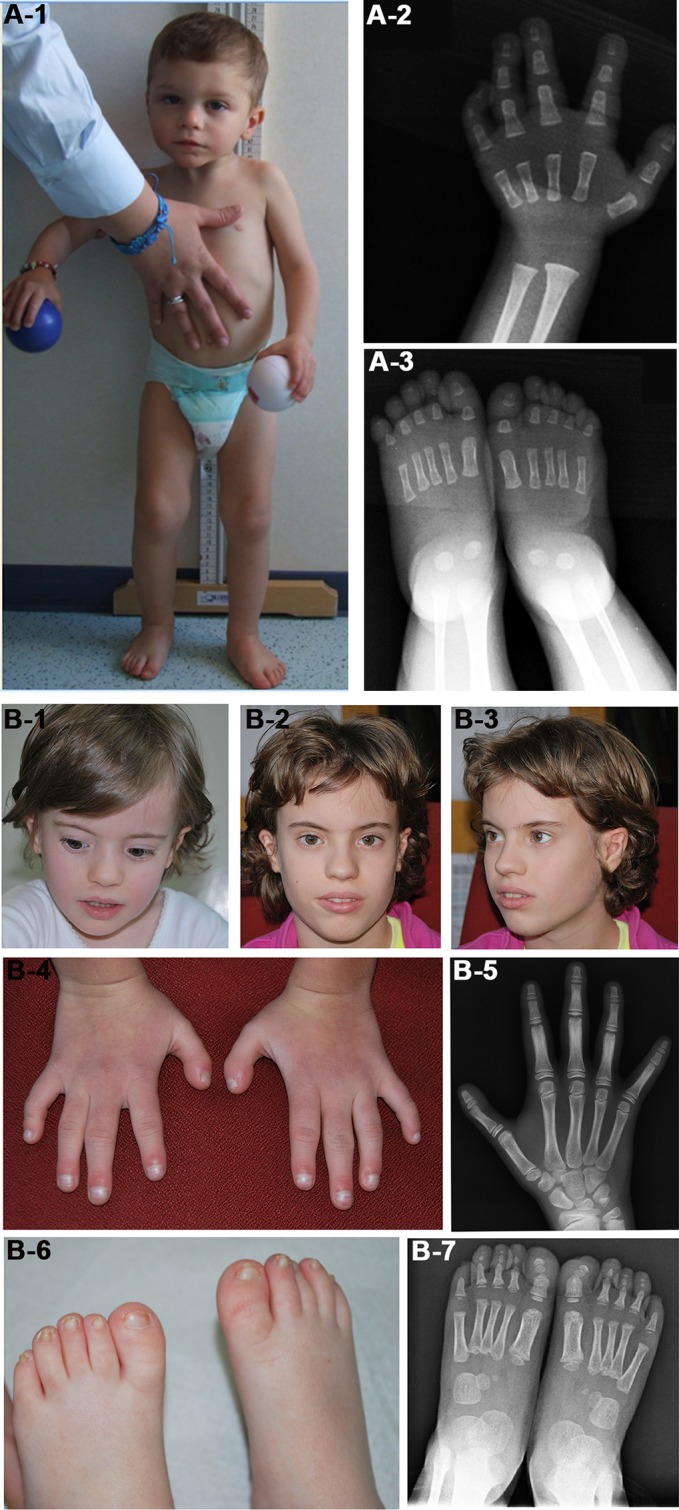
Photographs of patients 7 **(A)** and 20 **(B)**. **(A-1)** Face and whole body of patient 7 at 28 months showing proportionate short stature, mild craniofacial dysmorphisms, that is, triangular face and slight micrognathia, and flat feet. **(A-2 and 3)** Left hand and feet X-rays of the patient at 11 months showing **(A-2)** absence of the carpal bone nuclei, suggestive of a delayed bone age, fifth finger clinodactyly with hypoplastic and triangular shaped intermediate phalanx and distal phalanx hypoplasia, and **(A-3)** short first toes. The absence of bone nuclei at the carpus **(A-2)** and their presence at the tarsus **(A-3)** suggest a non-uniform pattern of skeletal maturation. **(B-1, B-2, and B-3)** Facial appearance of patient 20 at 4 years **(B-1)** and 10 years **(B-2, B-3)** showing prominent forehead and triangular face more evident in childhood. **(B-4)** Small hands with proximal placement and ulnar side bowing of first fingers, and clinodactyly of fifth fingers. **(B-5)** Left hand X-rays of the patient at 10 years showing hypoplasia of distal phalanges. **(B-6)** Small feet with short and broad halluces, right over-riding toes, and small/deep-set nails. **(B-7)** Feet X-rays of the patient at 4 years showing proximal placement of fifth metatarsals, absence of distal phalanges in second and fifth toes, absence of middle phalanges in fifth toes, and phalanges hypoplasia.

We identified in patient 7 a *de novo* deletion of 11 Mb at 1q, arr[GRCh37] 1q24.3q25.3(172652343_183538289)x1 dn, comprising 98 RefSeq genes (**Table 2**, **Figure 1A**). Deletions within the critical region 1q24q25 (**Figure 1A**) have been associated with a distinctive syndromic phenotype characterized by facial dysmorphism, small hands and feet with fifth finger clinobrachydactyly, severe proportionate short stature with microcephaly, and severe cognitive disability (Burkardt et al., 2011; Ashraf et al., 2015). Patient 7 was born preterm with cesarean section due to severe intrauterine growth restriction (IUGR) in the context of altered umbilical arterial flow and insufficient placental supply. In the first months of life, he had feeding difficulties, requiring gastric tube feeding (**Table 3**). At clinical evaluation at 28 months, he showed growth retardation, slight dolichocephaly, triangular face, slight micro-retrognathia, and teeth and genital abnormalities (**Table 3**, **Figure 2A**). He also displayed bilateral fifth finger clino/brachydactyly, flat feet, and delayed bone maturation, as revealed by a skeletal survey performed at 11 months (**Figure 2A**). Further medical problems included slight global developmental delay, attention deficit, and reduced social interaction. The small number of clinical features reminiscent of SRS is consistent with a clinical score of 3/6 (**Table 3**). Based on the partial overlap of the patient’s CNV with the 1q24q25 microdeletion syndrome critical region, the gene content of this region was taken into account in the re-evaluation of the overall patient’s phenotype.

Patient 17 was found to carry a *de novo* 230-kb duplication in 17p13.3 affecting five genes, arr[GRCh37] 17p13.3(1130776_1361490)x3 dn, mapping inside the region causative of the 17p13.3 duplication syndrome (OMIM#613215) and the Miller–Dieker syndrome (MDS, OMIM#247200) (**Table 2**, **Figure 1B**). The patient was born at term with slight growth restriction, which worsened in the next months when he also presented feeding difficulties (**Table 3**). His features are consistent with clinical SRS diagnosis: he fulfilled four out of six criteria and showed typical SRS facial appearance characterized by relative macrocephaly, triangular face, prominent forehead, and downturned mouth with thin lips. Further medical problems included hypotonia and muscular hypotrophy, typical of 17p13.3 duplication syndrome.

In patient 20, a *de novo* deletion of 7.5 Mb at 7p was detected, arr[GRCh37] 7p15.3p14.3(23236782_30690453)x1 dn, including 80 RefSeq genes (**Table 2**, **Figure 1C**). The patient was born at term and showed prenatal as well as postnatal growth restriction (**Table 3**). She had feeding difficulties and motor delay as she sat up alone at 9 months and walked autonomously at 17 months. Clinical evaluation at 4 years revealed relative macrocephaly, triangular face, and prominent forehead (**Figure 2B**). Her features satisfied four NH-CSS criteria; however, clinical SRS diagnosis cannot be confirmed, as she did not show relative macrocephaly at birth. Neuropsychological evaluation at age 10 using Wechsler Intelligence Scale for Children (WISC)-III scales (Wechsler, 2002) showed borderline intellectual functioning (QIT: 79; QIV: 78; and QIP: 85), as the girl needed a support teacher at high school. Her height improved from −3.16 SD (age 6 years) to −1.84 SD (age 10 years) in response to growth hormone (GH) therapy, and bone age assessment at 11 years showed a correspondence between bone age and chronological age. She also presented features not belonging to the SRS spectrum, such as small hands and feet with symmetric skeletal anomalies (**Figure 2B**), and duplex collecting system of the right kidney.

Segregation analysis from parents to proband of D7S493 and D7S2525 microsatellites, mapping inside the 7.5-Mb deletion, showed that the deletion occurred on the paternal chromosome (**Supplementary Figure 1**). In order to assess whether the *insulin-like growth factor 2 mRNA binding protein 3* (*IGF2BP3*) gene (OMIM*608259)—involved in the deletion and potential candidate for the patient’s phenotype—might be downregulated, gene expression analysis was performed. Real-time RT-PCR analysis confirmed the hypothesis, identifying a halved amount of *IGF2BP3* transcript in the patient compared with controls (**Supplementary Figure 1**). It is worth noting that patient 20 has two further deletions on proximal 7p, the first at 7p21.3 sized 81 kb and the second at 7p21.1 of 11 kb, both categorized as VOUS.

A *de novo* deletion of 1.15 Mb at 22q, arr[GRCh37] 22q11.2q11.22(21808950_22963000)x1 dn, encompassing 21 RefSeq genes, was identified in patient 23 (**Table 2**, **Figure 1D**). The deletion overlaps the critical region of distal 22q11.2 microdeletion syndrome (OMIM#611867), which is associated with a variable phenotype with prenatal growth delay. The girl was born preterm, at 35 weeks of gestation, and showed SGA, a triangular face, and fifth finger clinodactyly (**Table 3**). Growth retardation persisted at the age of 3 years. No cardiac defects or other features frequently associated with distal 22q11.2 microdeletion were present. The molecular findings did not confirm the initial clinical suspicion of SRS in keeping with the clinical presentation and the negative clinical score (2/6 items).

Lastly, in patient 28, who presents five NH-CSS criteria except for relative macrocephaly at birth (**Table 3**), we identified a deletion on chromosome 17q, arr[GRCh37] 17q22(57075470_57235248)x1 mat, of 159.8 kb, involving the *TRIM37*, *SKA2*, and *PRR11* genes (**Table 2**, **Figure 1E**). Mutations in the *tripartite motif containing 37* (*TRIM37*) gene (OMIM*605073) are causative of the MULIBREY nanism (OMIM#253250), a recessive syndrome with SRS overlapping clinical features (Hämäläinen et al., 2006). Indeed, *TRIM37* gene sequencing revealed a pathogenic single base change c.1949-12 A > G in intron 18 (NM_001005207) inherited from the patient’s healthy father (**Figure 1E**), as reported by Mozzillo et al. (2016).

### Potentially Pathogenic Variants (PP-CNV)

**Figure 3:**
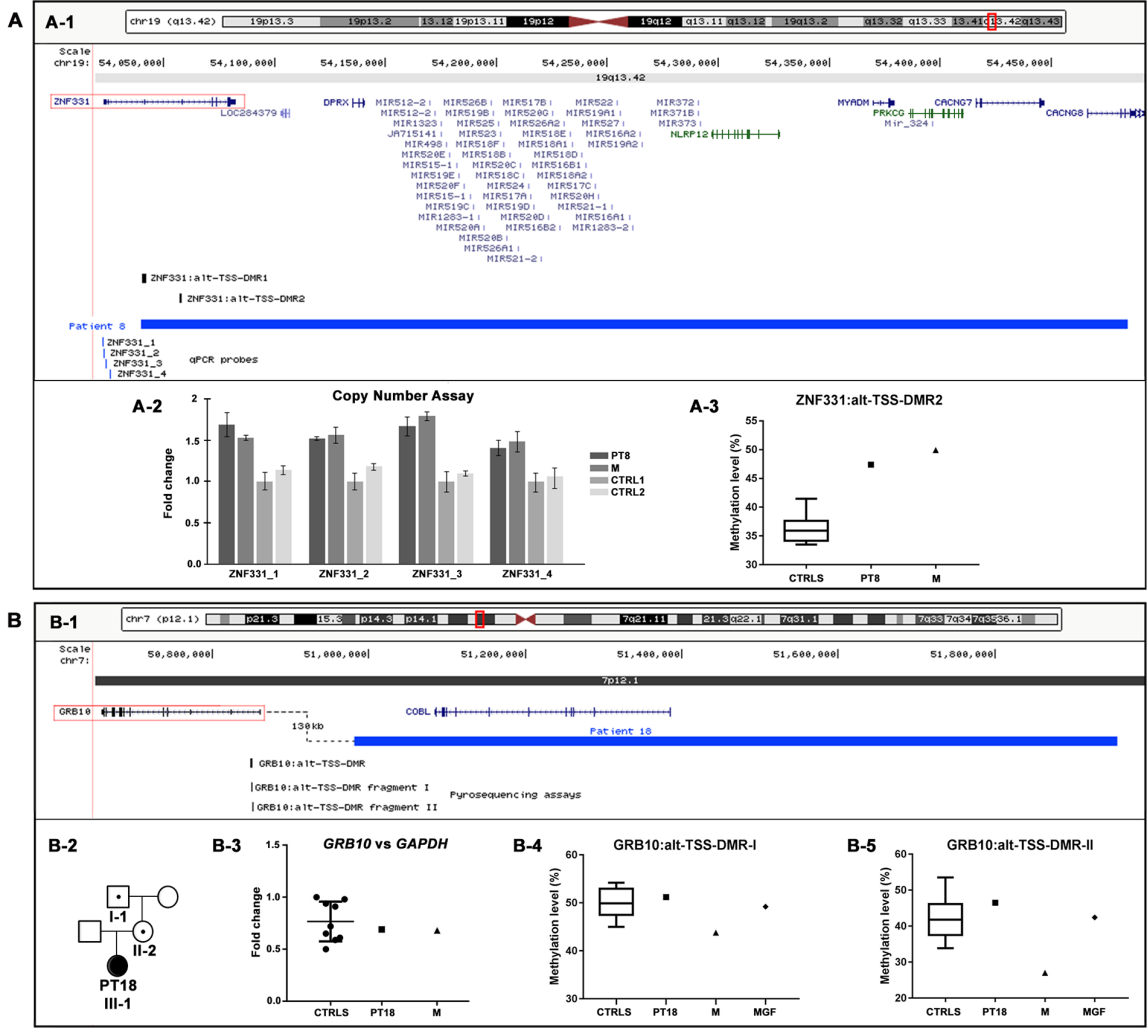
Physical map of the genomic regions and molecular characterization of the potentially pathogenic CNVs identified by array CGH. The 19q13.42 **(A-1)** and 7p12.1 **(B-1)** regions showing in blue the duplication identified in patients 8 **(A-1)** and 18 **(B-1)**. The RefSeq genes are depicted in dark blue, and the OMIM disease genes in green; the candidate genes are illustrated by red boxes, and the differentially methylated regions (DMRs) in black. The positions of pyrosequencing assays and qPCR probes used in the CNVs’ molecular characterization are reported in black and blue, respectively. The images are a modification of a version obtained from the UCSC Genome Browser (human genome assembly GRCh37/hg19). **(A-2)** Copy number analysis by using qPCR probes specific for *ZNF331* gene promoter confirmed the entire duplication of *ZNF331* gene in both patient 8 and her mother (M). **(A-3)** Pyrosequencing analysis performed on blood DNA of the ZNF331:alt-TSS-DMR2 showed an hypermethylation level in both patient 8 and her mother (M) compared with healthy controls. (B-2) Patient 18’s family tree showing duplication transmission. **(B-3)** Relative expression of *GRB10* blood mRNA in patient 18 and her mother (M), compared with healthy controls. Data were normalized against *GAPDH* as housekeeping gene; similar results were obtained using *TBP*, *HMBS*, and *RPLP0* as normalizer (data not shown). **(B**-**4**, **B**-**5)** Pyrosequencing analysis of the GRB10:alt-TSS-DMR performed on blood DNA showed a methylation level in patient 18 and her maternal grandfather (MGF) compared with healthy controls with both assays, while in patient 18’s mother (M), a hypomethylation level was detected with both assays. CNVs, copy number variants; CGH, comparative genomic hybridization; OMIM, Online Mendelian Inheritance in Man; qPCR, quantitative polymerase chain reaction; UCSC, University of California Santa Cruz.

Two CNVs were classified as PP variants on the basis of gene content and genomic position criteria. **Figure 3** shows the genomic regions containing the PP-CNVs of patients 8 and 18, as well as the integrated molecular analyses performed to assess their pathogenic effect.

Specifically, in patient 8, the aCGH analysis showed a duplication of 445 kb in 19q, arr[GRCh37] 19q13.42(54039784_54484439)x3 mat (**Table 2**, **Figure 3A-1**). The patient’s clinical data were insufficient for NH-CSS evaluation. She showed facial dymorphisms reminiscent of SRS and growth retardation during early childhood (**Table 3**). The proximal duplication breakpoint (bkp) lies within the *zinc finger protein 331* (*ZNF331*) gene (OMIM*606043) (isoform NM_018555), an imprinted gene yet unrelated to SRS. qPCR experiments clarified that the *ZNF331* gene and its two maternally methylated DMRs (ZNF331:alt-TSS-DMR1 and ZNF331:alt-TSS-DMR2) are fully duplicated (**Figure 3A-2**), and consistently, pyrosequencing analysis of DMR2 showed an increase of methylation in the patient and her mother as compared with controls (**Figure 3A-3**).

In patient 18, a duplication of 975 kb at 7p, arr[GRCh37] 7p12.1(50981149_51956510)x3 mat (**Table 2**, **Figure 3B-1**), was detected. The patient fulfills the NH-CSS criteria with a score of 4/5. No information about her head circumference at birth was available to establish a proper clinical SRS diagnosis. She did not show severe birth retardation, but psychomotor developmental delay and her facial dysmorphisms were not associated with SRS (**Table 3**). Her mother did not show any SRS features. The duplication fully involves the *cordon-bleu* (*COBL*) gene (OMIM*610317) and interestingly, its distal bkp maps 130 kb from the promoter of the *growth factor receptor-bound protein 10* (*GRB10*) gene (OMIM*601523), which is a candidate for SRS (Wakeling et al., 2017; Miyoshi et al., 1998). Further molecular analyses revealed that the duplication is transmitted by the phenotypically healthy grandfather (**Figure 3B-2**). Neither *GRB10* expression nor *GRB10*:alt-TSS-DMR methylation are impaired by the structural variant in the carrier family members (**Figures 3B-3–5**), with the exception of a slight hypomethylation detected in the mother compared with her daughter and controls (**Figures 3B-4 and 5**).

## Discussion

This study aimed to evaluate, through high-resolution aCGH, a cohort of 34 patients with clinical signs resembling SRS but negative to (epi)genetic tests. Five individuals, accounting for 15% of the starting cohort, were found to harbor pathogenic CNVs (P-CNVs), attesting a frequency higher than that shown by similar studies, ranging from 6% (Inoue et al., 2017) or 7% (Azzi et al., 2015) to 12% (Sachwitz et al., 2017). Furthermore, our detection rate increases to 21% by including two cases carrying PP-CNVs, whose exact pathogenic role awaits confirmation by further studies.

Regarding the NH-CSS, three out of five patients with P-CNVs (patients 17, 20, and 28) and one (patient 18) out of two with PP-CNVs fulfill the criteria, and one patient (patient 7) with P-CNV scored positive for three clinical items, reinforcing the recommendation to process by aCGH patients with a clinical suspicion of SRS. Only one patient (patient 17) can be diagnosed clinical SRS, while the other six patients, despite matching four or more criteria, are unlikely SRS.

Our results confirm the genetic heterogeneity found in similar studies (Tumer et al., 2018), increasing the number of structural variants identified to date in patients with SRS or similar features. The detected CNVs involve distinct genomic regions either previously associated with growth delay conditions (1q24.3-q25.3, 17p13.3, 17q22, and 22q11.2-q11.22) and with SRS spectrum (7p12.1 and 7p15.3-p14.3) or outlined for the first time (19q13.42), hence enhancing the definition of reported and novel SRS overlapping syndromes.

Patient 7 carries a P-CNV associated with 1q24q25 microdeletion syndrome (**Figure 1A**) and his phenotype, matching that of the described patients (Burkardt et al., 2011; Ashraf et al., 2015), recapitulates the SRS phenotype in terms of a few minor dysmorphisms and growth retardation. Three “key” genes for growth delay map within the deletion: *CENPL*, *centromere protein L* (OMIM*611503), involved in kinetochore function and mitotic progression (Burkardt et al., 2011); *PAPPA2*, *pappalysin 2*, encoding a protease for IGFBP3 and IGFBP5 involved in IGF-1 availability (Dauber et al., 2016), whose biallelic mutations account for short stature (Dauber et al., 2016); and *LHX4*, *LIM homeobox 4* (OMIM*602146) causative of the dominant pituitary hormone deficiency-4 syndrome (CPHD4; OMIM*262700).

The duplication at 17p13.3 of the clinically SRS patient 17 includes only five genes and to the best of our knowledge is the smallest reported to date. Two non-overlapping deletions of the same region have been identified in clinically suspected SRS patients (Spengler et al., 2012). Microduplications at 17p13.3, involving the *YWHAE* and *CRK* genes, similarly to our patient, are associated with macrosomia in the majority of cases (Henry et al., 2016). Conversely, patient 17 displays growth retardation and does not show the microduplication-associated neurologic dysfunctions, confirming the incomplete penetrance and highly variable expressivity of this duplication syndrome. Among the duplicated genes playing a role in cellular growth pathways, *CRK* (OMIM*164762) has been proposed as a candidate for body size determination (Østergaard et al., 2012).

Patient 23 was found to carry the distal 22q11.2 microdeletion syndrome (OMIM#611867), a recurrent rearrangement associated with prenatal and postnatal growth retardation (Ben-Shachar et al., 2008). She is unlikely SRS, though the same 1.1 Mb deletion has been reported in an SRS patient matching five NH-CSS criteria (Bruce et al., 2010), and an atypical distal 22q11.2 microdeletion has been described in a patient with clinical features reminiscent of SRS (Garavelli et al., 2011). The main candidate genes of this syndrome are *UBE2L3*, *ubiquitin conjugating enzyme E2 L3* (OMIM*603721), whose homozygous mutations in mice lead to placental defects and growth retardation (Harbers et al., 1996), and *MAPK1*, *mitogen-activated protein kinase 1* (OMIM*176948), encoding a protein kinase with an essential role in embryonic development (Saba-El-Leil et al., 2003).

Patient 28 was diagnosed to be affected by MULIBREY nanism (OMIM#253250), as he carries a 17q22 chromosome deletion and a concomitant *TRIM37* splicing mutation affecting the non-deleted chromosome, as already described (Mozzillo et al., 2016), reconfirming the clinical overlap between SRS and MULIBREY nanism (Hämäläinen et al., 2006; Jobic et al., 2017).

Finally, patient 20 showed a *de novo* deletion of 7.5 Mb on the paternal chromosome 7 at p15.3-14.3, proximal to the candidate 7p12 SRS region. She fulfills the NH-CSS except for body asymmetry, less common in upd(7)mat than in 11p15 LOM molecularly confirmed SRS patients (Wakeling et al., 2017), and relative macrocephaly at birth, although displayed in early childhood. Out of the numerous deleted genes that may account for SRS similar features, *IGF2BP3* is the most relevant, as it encodes for an RNA-binding factor specific to the 5′UTR of *IGF2* mRNA, regulating the *IGF2* transcript levels during fetal growth and development (Monk et al., 2002). A similar *IGF2BP3* deletion was found in an SRS patient with classical SRS phenotype, supporting the relevance of this *locus* in SRS etiology (Lin et al., 2010). Another interesting disease gene is *HOXA13*, *homeobox A13* (OMIM*142959), causative of the hand-foot-genital syndrome (HFGS, OMIM#140000), which is characterized by small feet with unusually short and big toes and abnormal thumbs, and urogenital malformations. This gene is also involved in heterogeneous chromosomal aberrations of the 7p15-p14 region, and like the reported cases, our patient shows feet and kidney anomalies (Devriendt et al., 1999; Dunø et al., 2004; Kosaki et al., 2005; Fryssira et al., 2011; Jun et al., 2011; Hosoki et al., 2012; Pezzani et al., 2015).

Regarding PP-CNVs, the maternal duplication at 19q13.42, identified in patient 8, comprises the chromosome 19 microRNA cluster (C19MC) and the *ZNF331* gene, whose ZNF331:alt-TSS-DMR2 was found to be hypermethylated in both patient and mother. *ZNF331*, encoding a zinc finger protein that acts as a tumor suppressor and negative regulator of cellular growth (Yu et al., 2013; Wang et al., 2013), was found to be differentially expressed between normal and IUGR placentas (Diplas et al., 2009). Up to now, data on maternal or paternal *ZNF331* expression are scarce and contradictory (Lambertini et al., 2012; Daelemans et al., 2010; Pollard et al., 2008; Pant et al, 2006), while the adjacent cluster of 46 miRNAs is paternally expressed in the placenta (Noguer-Dance et al., 2010). Recently, a paternally inherited duplication of 1.06 Mb, fully involving *ZNF331* and *C19MC*, was stated to be pathogenic in a fetus with IUGR (Petre et al., 2018) that in our opinion might be ascribed to the duplication of the paternally expressed C19MC cluster. The fetus’s father, in turn, inherited the duplication from his mother mirroring, our patient 8 and her mother, but his phenotype is not described. *ZNF331* is worth considering as a candidate gene for growth disorders, though further studies are needed to elucidate the pathomechanism.

A second maternal duplication at 7p12 was identified and categorized as PP-CNV in case 18 presenting a positive clinical score of 4 out of 5 (relative macrocephaly at birth is unknown). The CNV maps about 130 kb from the promoter of *GRB10* (**Figure 3B-1**), an imprinted gene with biallelic expression in many tissues, but with maternal expression in muscle and placenta (Blagitko et al., 2000; Monk et al., 2009). Genomic imbalances affecting *GRB10*, in particular, maternal duplications involving *GRB10*, lead to SRS-like features and growth retardation (Tumer et al., 2018; Yuan et al., 2016). Since the duplication is inherited from the healthy maternal grandfather *via* the healthy mother, an imprinting transmission pattern might be envisaged. Even if concordant results from both expression and methylation analyses on family member blood cells did not support this hypothesis, the pathogenic role of the duplication remains challenging as a perturbed gene regulation cannot be ruled out during the fetal development, in particular in tissues where the gene is only maternally expressed.

In conclusion, we found five clinically relevant CNVs and two VOUS—PP-CNVs in distinct chromosomal regions in 15% and 6% of clinically suspected SRS patients, respectively. Out of these variants, two, harbored by patients matching the NH-CSS criteria, map to 7p distinct regions, reinforcing the contribution of the short arm of chromosome 7 to SRS-like etiology. Further investigations on a larger cohort of SRS/SRS-like patients are needed, firstly, to confirm the observed relevant CNVs detection rate, to exclude coincidental findings unrelated to the phenotype of the reported patients, and to support the role of the appointed genes in SRS and similar conditions.

Genome-wide aCGH scan integrated by in-depth studies is re-confirmed as an appropriate and powerful tool for achieving a differential diagnosis in both clinically and unlikely SRS diagnosed patients and to highlight novel chromosome alterations and genomic regions associated with SRS and similar growth retardation disorders.

## Data Availability Statement

The datasets generated for this study can be found in ClinVar database (http://www.ncbi.nlm.nih.gov/clinvar/), accession numbers SCV000863548 to SCV000863554.

## Ethics Statement

The study was approved by the ethical committee of Istituto Auxologico Italiano and written informed consent was obtained from the parents for publication of this study and any accompanying images.

## Author Contributions

MC: study design, analysis, and interpretation of clinical and experimental data, and manuscript preparation; MB: clinical data collection and interpretation; LC: performed preliminary diagnostic methylation-specific multiplex ligation-dependent probe amplification (MS-MLPA) analysis and collection of clinical data; CP: analysis and interpretation of data from aCGH; CG: performed pyrosequencing analysis; ASi: performed qPCR analysis; IB: analysis and interpretation of data from aCGH and CNVs submission to ClinVar; SG: performed preliminary diagnostic MS-MLPA analysis; ASe, AM, and SB: clinical data collection; AR: critical revision of the manuscript; GF: clinical data collection; SR: clinical data collection and critical revision of the manuscript; LL: manuscript preparation and critical revision of the manuscript; PF: study design, interpretation of experimental data, manuscript preparation, and critical revision of the manuscript.

All authors read and approved the manuscript for submission.

## Funding

This study was partially supported by the Italian Ministry of the University and Research PRIN grants 2009 and 2015 (Prot. 2009MBHZPR_004 and Prot. 2015JHLY35) to PF and by Italian Ministry of Health grant “Ricerca Corrente” to IRCCS Istituto Auxologico Italiano (PF 08C301-2013).

## Conflict of Interest

The authors declare that the research was conducted in the absence of any commercial or financial relationships that could be construed as a potential conflict of interest.

The reviewer TE declared a past co-authorship with several of the authors AR, LC, SG, AS, AM, SR to the handling editor.
