# Supplementary material for: Molecular Etiology Disclosed by Array CGH in Patients With Silver–Russell Syndrome or Similar Phenotypes
Source: Front Genet. 2019 Oct 15;10:955. doi: 10.3389/fgene.2019.00955 (PMC6843062; doi:10.3389/fgene.2019.00955)
Supplement: Supplementary file 1 [file Table_1.docx]

**Supplementary table 1. Primers used in qPCR analysis**

| ***Probe*** | *Designation* | ***Primer sequence (5’🡪3’)*** | ***Primer localization^a^*** | ***Genomic position*** |
| --- | --- | --- | --- | --- |
| *ZNF331_1* | ZNF331-5FW | ATCCAGTCACACCCACCAAT | chr19:54022485-54022504 | Upstream *ZNF331* (NM_018555) |
|  | ZNF331-5RW | TGAGATGCTTTGCCCTAAAAA | chr19:54022531-54022551 |  |
| *ZNF331_2* | ZNF331-6FW | GAGAGAGCCTTGGCACAGAC | chr19:54022994-54023013 | Upstream *ZNF331* (NM_018555) |
|  | ZNF331-6RW | GGCATGTGTGTAGGGACCTT | chr19:54023050-54023069 |  |
| *ZNF331_3* | ZNF331-7FW | GGATTTCCGGGTTATGGACT | chr19:54024125-54024144 | *ZNF331* exon 1 (NM_018555) |
|  | ZNF331-7RW | CAACTCTACACGGCGCAAA | chr19:54024177-54024196 |  |
| *ZNF331_4* | ZNF331-8FW | GGTCCTGGTGCCTCATTCT | chr19:54025827-54025846 | *ZNF331* IVS 1 (NM_018555) |
|  | ZNF331-8RW | CGGGATGCTTTTCCTGAAG | chr19:54025875-54025894 |  |
| *Pt20qPCR_1* | Pt20qPCR-1F | TATGCCAAGATTCCGGTAGG | chr7:20819780-20819799 | Inside 7p21.1 pt’s deletion |
|  | Pt20qPCR-1R | GATGCTGGAAAAAGCTCCTG | chr7:20819828-20819847 |  |
| *Pt20qPCR_2* | Pt20qPCR-2F | GAAGAGGGGCAGAAACAGAA | chr7:20823771-20823790 | Inside 7p21.1 pt’s deletion |
|  | Pt20qPCR-2R | TCAACTCCAGCCTTTCCCTA | chr7:20823817-20823836 |  |
| ^a^Primer physical localization is based on GRCh37/hg19 human genome assembly. | | | |  |
