# Supplementary material for: Molecular Etiology Disclosed by Array CGH in Patients With Silver–Russell Syndrome or Similar Phenotypes
Source: Front Genet. 2019 Oct 15;10:955. doi: 10.3389/fgene.2019.00955 (PMC6843062; doi:10.3389/fgene.2019.00955)
Supplement: Supplementary file 2 [file Table_2.docx]

**Supplementary table 2: Oligonucleotides and amplification conditions used to perform *TRIM37* mutational screening**

| ***Designation*** | ***Primer sequence (5’🡪3’)*** | ***Annealing T(°C)*** | ***PCR size (bp)*** |
| --- | --- | --- | --- |
| *TRIM37-1F*  *TRIM37-1R* | CTGGACCTGAAGAGATGGGG  CTACTGGAGGGAGGACATGG | 61.3ºC | 238 bp |
| *TRIM37-2F*  *TRIM37-2R* | TCTTGACCAACCAAATCCTGA  CAAGCAAGCACTTTAGGGCA | 56.5°C | 360 bp |
| *TRIM37-3F* | TGACAGTGAGGGAAACAGTAGA | 57.2ºC | 632 bp |
| *TRIM37-3R* | TCACATGAATTAGTCCCTGCAT |  |  |
| *TRIM37-4F* | CAAGGCTACAGTGAACCATGA | 56.5ºC | 399 bp |
| *TRIM37-4R* | ACAGCGCATGACTGAAAGAA |  |  |
| *TRIM37-5F* | AACTGACCTGTTTGCCCAAG | 56.5ºC | 219 bp |
| *TRIM37-5R* | GTCCATCAAACCACACAAACA |  |  |
| *TRIM37-6F* | CACTGGCCTGCTTCAACAAA | 56.7ºC | 344 bp |
| *TRIM37-6R* | GGTACAGCCTATCAAATTTGCA |  |  |
| *TRIM37-7F* | TCATTTCCATTTGACCTTCCTCT | 57.2ºC | 343 bp |
| *TRIM37-7R* | GCTACCTAACAATGATGCCCA |  |  |
| *TRIM37-8F* | GAAGTTCCATTTTCAAGGCTACT | 57ºC | 313 bp |
| *TRIM37-8R* | AGGCAGGAGAATGGTGTGAA |  |  |
| *TRIM37-9F* | AGCTTTCAACTTACCGGTTTGT | 57.4ºC | 391 bp |
| *TRIM37-9R* | AGAAAAGAGAGAGAGAGATGGCA |  |  |
| *TRIM37-10F* | CCACCTCACCCAGCCAAAA | 59.3ºC | 385 bp |
| *TRIM37-10R* | TCCCAGACTAGAGAAGCAGTC |  |  |
| *TRIM37-11F* | ACAGTGGAATTTTGACCTGTTCA | 58ºC | 231 bp |
| *TRIM37-11R* | GGAAGAAGGGGAACAGGGAA |  |  |
| *TRIM37-12F* | TCCATTGCTTTGAGGCTTAGA | 55.3ºC | 248 bp |
| *TRIM37-12R* | ACGCAAAGCAATACAAGTTTTCT |  |  |
| *TRIM37-13F* | GCGTATATCAATAAGGTTTCTTCTCA | 56.6ºC | 354 bp |
| *TRIM37-13R* | TCTGATGATATTATTTCCCCACAA |  |  |
| *TRIM37-14F* | CCCAAATTGCAGAATTCAGTG | 56.5ºC | 348 bp |
| *TRIM37-14R* | TGGTAAGGATGGGGAACAAG |  |  |
| *TRIM37-15F* | GGGCAACAGAGTGAGACCAT | 58ºC | 428 bp |
| *TRIM37-15R* | TGTTAAAAGCCATGATGCTACTG |  |  |
| *TRIM37-16F* | GAGGCACAGGAGCAGAAAAT | 57.2ºC | 425 bp |
| *TRIM37-16R* | ATTGGCTAAGAGCCCAGAGA |  |  |
| *TRIM37-17F* | TGACAAAAATCTTTCTCCTTTTTG | 54.5ºC | 342 bp |
| *TRIM37-17R* | TCCAACACCATGAACCATGT |  |  |
| *TRIM37-18F* | GCCCTGGTTCCTTCTTTCTG | 58.5ºC | 448 bp |
| *TRIM37-18R* | CCATGTTCCACACATAGCTGA |  |  |
| *TRIM37-19F* | TGGAATTTAGTGCATGTTTTATTG | 52.4ºC | 606 bp |
| *TRIM37-19R* | CGGAATCAATTTTGGATGAA |  |  |
| *TRIM37-20F* | CCAATTGGGGCTACAGAATG | 58.3ºC | 440 bp |
| *TRIM37-20R* | AGCCTGGGTGACAAAGTGAG |  |  |
| *TRIM37-21F* | CAGGCTGGAGTGTAGCACAA | 58.4ºC | 411 bp |
| *TRIM37-21R* | GCATACTTAAAGTTCCAAACAAAGC |  |  |
| *TRIM37-22F* | CCAGAGCACGTACCCTACTT | 59.1ºC | 411 bp |
| *TRIM37-22R* | CCACCACCCACCACCAAAA |  |  |
| *TRIM37-23F* | ATCGAGGTTTGTGCGGTTAC | 56.5ºC | 414 bp |
| *TRIM37-23R* | GAAGCAGAAGAAGCAAAAGCA |  |  |
| *TRIM37-24F* | TTGTTCACAACCCAGCACAT | 56.2ºC | 379 bp |
| *TRIM37-24R* | AAACTGTGCCACCTTCCAAC |  |  |
| *TRIM37-25F* | CCCACTGAGAAAGCCTAGCA | 58.3ºC | 326 bp |
| *TRIM37-25R* | ATAGTTGGGGCCATGTTGAC |  |  |
