# Supplementary material for: Molecular Etiology Disclosed by Array CGH in Patients With Silver–Russell Syndrome or Similar Phenotypes
Source: Front Genet. 2019 Oct 15;10:955. doi: 10.3389/fgene.2019.00955 (PMC6843062; doi:10.3389/fgene.2019.00955)
Supplement: Supplementary file 3 [file Table_3.docx]

**Supplementary table 3. Primers used in Pyrosequencing analysis**

| ***Assay*** | ***Designation*** | ***Primer sequence (5’🡪3’)*** | ***Primer localization^a^*** |
| --- | --- | --- | --- |
| ZNF331:alt-TSS-DMR2 | ZNF331-2FW | AGAGGGTGTGGGGTGACGTA | chr19:54058016-54058036 |
|  | ZNF331-2RW | bio5’CCCGCCACTCCACACAATA | chr19:54058210-54058229 |
|  | ZNF331-2seq | TAGGGTTGGGGTGTT | chr19:54058055-54058069 |
| GRB10:alt-TSS-DMR fragment I | GRB10-1FW | TGTAGTAGTTYGGATGTTTAGA | chr7:50849713-50849734 |
|  | GRB10-1RW | bio5' CAAACTACAAAAACCCCRAC | chr7:50850015-50850034 |
|  | GRB10-1seq | GTTTAGATTTATTTATTTT | chr7:50849728-50849746 |
| GRB10:alt-TSS-DMR fragment II | GRB10-2FW | GGTTTTGGAGTATAATAGGAATT | chr7:50850569-50850591 |
|  | GRB10-2RW | bio5’ TCAAACAAAGRACATCTCTAAC | chr7:50850851-50850872 |
|  | GRB10-2seq | TTGGTTATTTTGATGAT | chr7:50850763-50850779 |
