# Supplementary material for: Molecular Etiology Disclosed by Array CGH in Patients With Silver–Russell Syndrome or Similar Phenotypes
Source: Front Genet. 2019 Oct 15;10:955. doi: 10.3389/fgene.2019.00955 (PMC6843062; doi:10.3389/fgene.2019.00955)

Supplementary Figure S1: Molecular characterization of the pathogenic CNV identified in patient 20. (A) Electropherogram of D7S493 and D7S2525 microsatellites on chromosome 7p, mapping inside the 7.5 Mb *de novo* deletion identified in patient 20, revealing the paternal origin of the deleted allele of patient 20. **(B)** Relative expression of *IGF2BP3* blood mRNA in patient 20, showing an halved amount of *IGF2BP3* transcript compared to healthy controls. Data were normalized against *GUSB* as housekeeping gene; similar results were obtained using *GAPDH* and *TBP* as normalizer (data not shown)

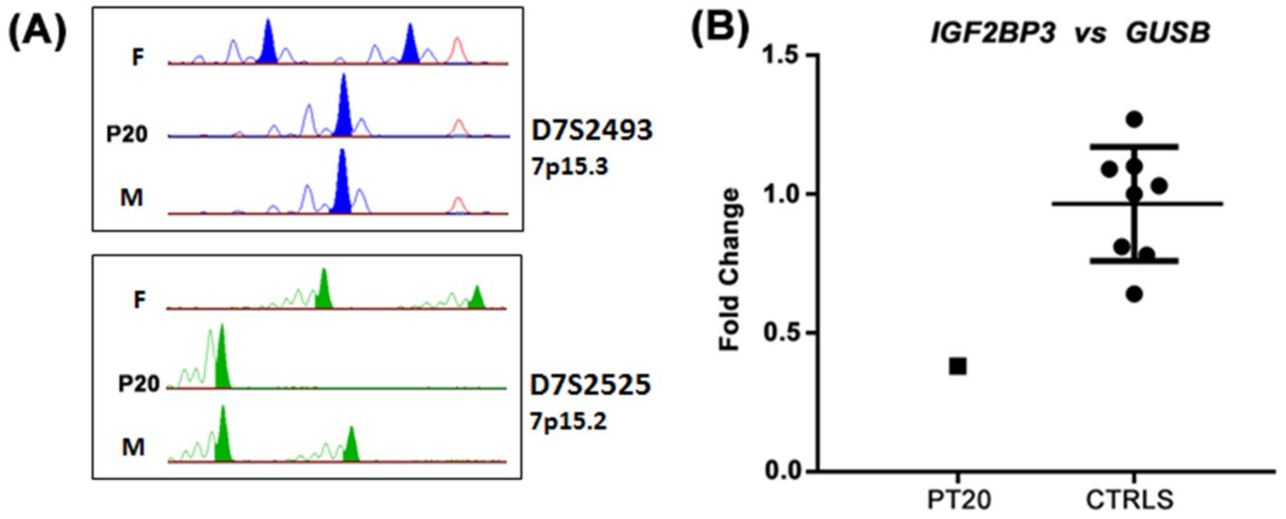

Supplement: Supplementary file 5 [file DataSheet_1.pdf]
